# The solid progress of nanomedicine

**DOI:** 10.1007/s13346-020-00743-2

**Published:** 2020-03-05

**Authors:** João Pedro Martins, José das Neves, María de la Fuente, Christian Celia, Helena Florindo, Nazende Günday-Türeli, Amirali Popat, José Luis Santos, Flávia Sousa, Ruth Schmid, Joy Wolfram, Bruno Sarmento, Hélder A. Santos

**Affiliations:** 1 Drug Research Program, Division of Pharmaceutical Chemistry, Faculty of Pharmacy, University of Helsinki, FI-00014 Helsinki, Finland; 2 i3S – Instituto de Investigação e Inovação em Saúde & INEB – Instituto de Engenharia Biomédica, University of Porto, Porto, Portugal; 3 Nano-Oncology Unit, Health Research Institute of Santiago de Compostela (IDIS), Clinical University Hospital of Santiago de Compostela (CHUS), CIBERONC, 15706 Santiago de Compostela, Spain; 4 Department of Pharmacy, University of Chieti – Pescara “G. d’Annunzio”, Chieti, Italy; 5 Research Institute for Medicines (iMed.ULisboa), Faculty of Pharmacy, Universidade de Lisboa, Lisbon, Portugal; 6 MyBiotech GmbH, Industriestr. 1B, 66802 Überherrn, Germany; 7 School of Pharmacy, The University of Queensland, Brisbane, 4102 Australia; 8 Dosage Form Design and Development, AstraZeneca, Gaithersburg, MD USA; 9 Department of Biotechnology and Nanomedicine, SINTEF Industry, Trondheim, Norway; 10 Department of Biochemistry and Molecular Biology, Mayo Clinic, Jacksonville, FL USA; 11 Helsinki Institute of Life Science (HiLIFE), University of Helsinki, FI-00014 Helsinki, Finland

**Keywords:** Nanomedicine, Nanotechnology, Clinical translation, Reproducibility, Cancer therapy

## Abstract

This commentary article conveys the views of the board of the Nanomedicine and Nanoscale Delivery Focus Group of the Controlled Release Society regarding the decision of the United States National Cancer Institute (NCI) in halting funding for the Centers of Cancer Nanotechnology Excellence (CCNEs), and the subsequent editorial articles that broadened this discussion.

**Figure:**
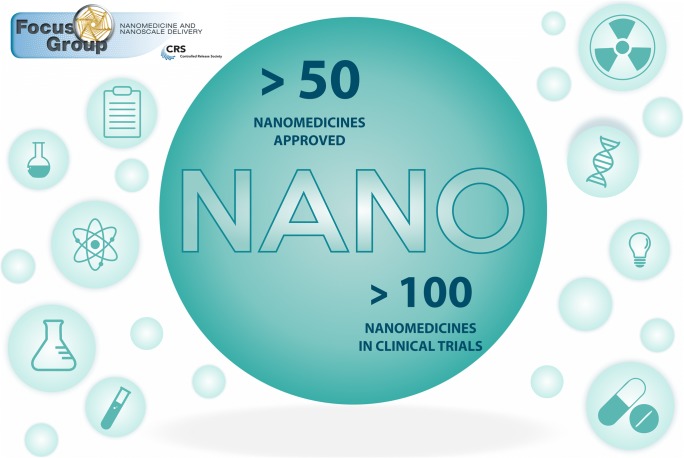
Graphical abstract

Graphical abstract

On May 2019, the journal *Science* reported that the United States National Cancer Institute (NCI) would halt funding for the Centers of Cancer Nanotechnology Excellence (CCNEs) [1]. This decision of the NCI triggered news headlines and was followed by an impactful commentary piece on nanomedicine, authored by Kinam Park, the former Editor-in-Chief of the Journal of Controlled Release [2]. Park conveyed that the decision was timely and represented the “beginning of the end” of the nanomedicine hype, laying out a series of arguments to support his statement. In a follow-up letter to the editor of the same journal [3], Piotr Grodzinski explained that the NCI uses a pool of “set aside” funds to support, for a limited period of time, the growth of emerging fields. This financial support is intended to make the field strong enough and, if worthy of investment, capable of competing via other funding mechanisms [3]. The NCI “set aside” funds supported the CCNEs for 15 years, during which two judicious decisions of renewal were followed by gradual budget cutbacks. The decrease in NCI funding to the CCNEs has been accompanied by a global growth in cancer nanotechnology research, resulting in a more than twofold increase in the number of cancer nanotechnology-related grant applications awarded worldwide between 2008 and 2018, as reported by Grodzinski [3]. Now that the field has matured enough, it is time for the anticipated non-renewal of the NCI financial support of the CCNEs under this program. In view of these events, we would like to take this opportunity to communicate the views of the board of the Nanomedicine and Nanoscale Delivery Focus Group of the Controlled Release Society.^1^

An extensive body of evidence has demonstrated the ability of several nanomedicines (both non-targeted and targeted) to increase active payload concentrations at the target site (e.g., tumor) [4–6], as well as to reduce toxicity and enhance therapeutic efficacy compared with free drugs in preclinical studies [7–9]. More importantly, studies in humans support the ability of nanoparticle-based therapies to enhance active payload accumulation in tumors, and to improve safety and/or anticancer efficacy [10–12]. Over the years, many nanomedicines have received clinical approval based on improved safety with equivalent efficacy. For example, Doxil® (liposomal doxorubicin) was approved for multiple myeloma due to a better safety profile compared with free doxorubicin [13]. In terms of therapeutic efficacy in clinical studies, there are also several nanomedicines that outperform their free drug counterparts. For example, in some phase III clinical trials for breast cancer, Abraxane® (albumin-bound paclitaxel nanoparticle) was shown to cause better treatment responses compared with free paclitaxel [14, 15]. Another example is the approval of Vyxeos® (liposomal daunorubicin and cytarabine); a phase III clinical trial for high-risk acute myeloid leukemia (AML) demonstrated that Vyxeos® resulted in a median overall survival of 9.56 months compared with 5.95 months with free cytarabine and daunorubicin combination therapy (standard of care) [16]. In addition, nanotechnology-enabled cancer therapies do not merely focus on placing drugs at the tumor site, but also seek to provide novel therapeutic approaches in line with the discovery of new disease mechanisms and the precision oncology concept, and to restrict the interplay with other non-tumor cells involved in tumor progression and dissemination [17, 18]. Hence, an improved understanding of the disease mechanisms will enable the development of more efficient nanomedicines with mechanisms of action beyond tumor nanoparticle accumulation [19, 20]. For instance, nanoparticles are currently being explored in the fields of adoptive cell therapy and immune modulation in various stages of preclinical and clinical development [21]. Moreover, recent preclinical studies and clinical trials have shown benefits of combination therapies, and particularly, the ability of nanoparticles to simultaneously deliver therapeutic agents, such as small molecules, genetic material, and biologics [22].

A careful analysis of the current nanomedicine market and development pipeline leaves little margin to question the value proposition that nanomedicines already play in healthcare. There are currently over 50 nanomedicines and nanotechnology-based medical products approved by regulatory bodies worldwide for a variety of indications [23–25]. Some additional examples of nanomedicines used in cancer therapy include Onivyde® (liposomal irinotecan) or Hensify® (hafnium oxide nanoparticles). There are also many nanomedicines that are used for indications other than oncology, such as the “classic” AmBisome® (liposomal amphotericin B) for fungal infections, or the recently approved Onpattro® (small interfering RNA-lipid nanoparticles) for hereditary transthyretin amyloidosis (ATTR). The latter constitutes the first-in-class RNA interference (RNAi) therapeutic, paving the way for many novel nanotechnology-based gene silencing therapeutics [26]. Additionally, an estimated 100 nanoparticle-based products are in clinical trials [24, 27], of which 18 started in the past 3 years, legitimating the idea that “the interest and pursuit of successful nanoparticle technologies continues,” highlighted by Anselmo and Mitragotri in their most recent update on nanoparticles used in clinical practice [28]. Indeed, many companies have been actively developing nanomedicines over the past years, and investing billions of dollars, either in developing their own pipeline or through acquisitions. These include small- to mid-sized firms focused on research and development (R&D), as well as multinational companies like Pfizer, Eli Lilly and Company, Novartis, and Sanofi, to name a few. Moreover, a promising shift within the nanomedicine research community is the additional focus on cardiovascular [29], autoimmune [30, 31], neurological [32], infectious [33], and genetic and rare diseases [34]. RNA-based synthetic vaccines are another emerging area with high potential for nanomedicine [35–37]. Hence, public and private science funders and policy makers should drive such diversification to stimulate the pursuit of nanomedicines for clinical applications beyond cancer.

Challenges involved in the clinical translation of nanomedicines include the lack of batch-to-batch reproducibility, long-term stability of some products, complexity of the manufacturing processes, and maintenance of sterile conditions. There is also a lag between continuous scientific advances and regulatory guidance, namely regarding the specific requirements that are necessary for nanomedicine products to advance for clinical trials. In addition, the lack of appropriate controls and poorly defined critical quality attributes, as well as the absence of clinically relevant animal models that truly recapitulate the mechanisms of action of nanomedicines in humans, have prevented widespread clinical translation [38, 39]. The limitations imposed by too simplistic approaches or too complex models that hinder reliable interpretation of data call attention to the need for standardization and stratification of methodology [40]. The gradual implementation of universally standardized practices could promote more accurate reporting of materials and methodologies, and could change the paradigm for many nanotechnology products that already exist [41, 42]. On top of this, improving the clinical impact of nanomedicines demands “smart thinking and rational and realistic reasoning,” as stated by van der Meel et al. in a recently published perspective article in Nature Nanotechnology [43]. In this sense, and particularly in the case of cancer therapy, patient stratification, rational drug selection, the use of combination therapies, and the targeting of the adaptive immune system are key for addressing scientific and medical questions that will potentiate the exploitation and, most importantly, the translation of nanomedicines into the clinic. As stated in Park’s commentary, “meaningful work” on nanomedicine rather than “focusing on publications” needs to be prioritized. However, this is not an idiosyncrasy of the nanomedicine field, but rather a transversal problem of scientific endeavors in general. In an ideal scenario, academic institutions should be efficiently working together with industry partners and regulatory agencies to bring innovative nanomedicines to clinical practice.

In a recent editorial in the journal *Nature Biomedical Engineering* titled “Targeting for delivery,” the clinical translation of cancer nanomedicines was addressed, and researchers were designated as those that “feel that there is a ‘delivery problem’” and those “who are optimistic” [44]. In light of the facts outlined in the present commentary article, the board of the Nanomedicine and Nanoscale Delivery Focus Group of the Controlled Release Society falls into the second group. Delivery systems are continuously evolving with an increased understanding of complexities of human diseases like cancer. We have grounds for optimism and reasons to allude to the Gartner hype cycle [45]. As for any other potential breakthrough, the disillusionment around nanomedicines for cancer applications is not more than a natural state after a “peak of inflated expectations.” However, the outcomes start to crystallize, and the “plateau of productivity” seems more realistic than a foreseeable ambition [46].

Despite the fact that several early promises of nanomedicine are still left unmet, the solid contributions of nanotechnology to the cancer therapeutics and diagnostics, and more generally on human health, cannot be ignored [46]. This is not the “beginning of the end,” but the turnaround from academic development and preclinical studies to systematic and translational approaches, industrial development, and clinical trials. We believe that nanomedicine as a research field is not languishing or doomed. On the contrary, as the body of fundamental knowledge on the complex interactions between nanomaterials and host increases, the likelihood of additional and innovative nanotechnology-based products being developed and approved also increases. By bringing clinicians, scientists, regulatory bodies, and the pharmaceutical industry working more closely together, this new era is likely to realize the full potential of nanomedicine and further revolutionize the healthcare system for the treatment of complex, rare, and incurable diseases.

## Footnotes

1 https://www.controlledreleasesociety.org/focus-groups/nanomedicine-and-nanoscale-delivery-nnd
